# Genome Instability Induced by Low Levels of Replicative DNA Polymerases in Yeast

**DOI:** 10.3390/genes9110539

**Published:** 2018-11-07

**Authors:** Dao-Qiong Zheng, Thomas D. Petes

**Affiliations:** 1 Ocean College, Zhejiang University, Zhoushan 316021, Zhejiang, China; 2 Department of Molecular Genetics and Microbiology, Duke University School of Medicine, Durham, NC 27710, USA

**Keywords:** DNA replication stress, genome instability, DNA polymerase α, DNA polymerase δ, DNA recombination

## Abstract

Most cells of solid tumors have very high levels of genome instability of several different types, including deletions, duplications, translocations, and aneuploidy. Much of this instability appears induced by DNA replication stress. As a model for understanding this type of instability, we have examined genome instability in yeast strains that have low levels of two of the replicative DNA polymerases: DNA polymerase α and DNA polymerase δ (Polα and Polδ). We show that low levels of either of these DNA polymerases results in greatly elevated levels of mitotic recombination, chromosome rearrangements, and deletions/duplications. The spectrum of events in the two types of strains, however, differs in a variety of ways. For example, a reduced level of Polδ elevates single-base alterations and small deletions considerably more than a reduced level of Polα. In this review, we will summarize the methods used to monitor genome instability in yeast, and how this analysis contributes to understanding the linkage between genome instability and DNA replication stress.

## 1. Introduction

The accurate duplication of genetic material is essential for life, and three B-family DNA polymerases (Pol α, δ, and ε) are critical for genome replication in the yeast *Saccharomyces cerevisiae*, as in other eukaryotes [1]. The roles of these three polymerases are different. Polα generates short DNA fragments by extending RNA primers at the replication fork, and Polδ and Polε synthesize the majority of the nascent lagging and leading strands, respectively, by extension of the Polα-generated fragments [2]. Exogenous (various chemicals, UV, and X-rays) and endogenous (cellular metabolites, defects in DNA polymerase, inactivation of DNA repair pathways) agents that interrupt the DNA replication process lead to genome instability [3,4]. By generating genetic diversity, genome instability contributes to tumorigenesis [5]. On the other hand, a very high rate of genome mutations may overwhelm DNA repair capability, leading to cellular senescence [6]. Therefore, understanding the molecular basis of genome instability and how certain genomic alterations affect phenotypes is crucial to the development of novel strategies for cancer diagnostics and treatment. Using yeast models in which the levels of DNA polymerases were reduced, our observations provided novel insights into how DNA replication stress stimulates DNA lesions, chromosomal recombination, and global genome instability.

In addition to elevating instability throughout the genome, drugs that reduce the levels of nucleotide pools or inhibit replicative DNA polymerases elevate double-stranded DNA breaks (DSBs) at specific loci called “fragile sites”. In mammalian cells, fragile sites share a number of properties. They tend to be regions of the genome that are late-replicating within large actively transcribed genes [7]. Fragile sites are preferred sites for the integration of viruses and are associated with the formation of deletions in tumor cells. Based on a variety of observations, it has been suggested that, under conditions of replication stress, regions that are normally late-replicating experience stalled replication forks that are at risk for formation of a double-stranded DNA break [7].

Although fewer studies have been done in the yeast *S. cerevisiae* compared to mammalian cells, breakage-prone sequence motifs have been identified both in normally dividing cells and in cells undergoing replication stress. One property in common among many of these motifs is their tendency to stall replication forks, likely related to their ability to form secondary DNA structures (hairpins, triplex DNA, G-quadruplexes) [8]. For example, both tracts of the trinucleotide CTG (capable of forming hairpin structures) and GAA tracts (associated with triplex formation) result in elevated levels of double-strand breaks and hyper-recombination [9,10,11]. Lastly, as described below, regions that are preferred sites for recombinogenic lesions under conditions of replication stress often co-localize with sites at which replication forks are slowed, or stalled, even under normal growth conditions [12,13].

## 2. Analysis of Genome Instability in Yeast

### 2.1. Commonly Used Assays of Genome Instability

Different assays are required to detect different types of genome instability. One assay commonly used to detect single-base substitutions and small insertions/deletions (in/dels) is to monitor the rate of mutations at the *CAN1* locus [14]. Strains with the wild-type *CAN1* gene (encoding an arginine permease) are sensitive to the arginine analogue canavanine. By measuring the frequencies of canavanine-resistant derivatives of these strains and converting those frequencies into rates using the method of the median [15] or related methods, one can obtain a rate of mutations for this gene. A similar method can be used to measure the rate of mutations within the *URA3* gene, since strains with a wild-type gene are poisoned by 5-fluoro-orotate [16]. Sequence analysis of the mutant genes is necessary to identify the nature of the mutation. In wild-type strains, most mutations in *CAN1* or *URA3* are single-base substitutions, but mutant strains or genes with high-GC content sometimes have a different spectrum of mutations [13,17,18].

A more laborious, but less restricted method, of measuring the rates and types of small alterations, is whole-genome sequencing [19,20]. Due to the low rate of unselected events in most genetic backgrounds, such studies often require sequencing many lines subcultured for many (>500) generations.

**Figure 1:**
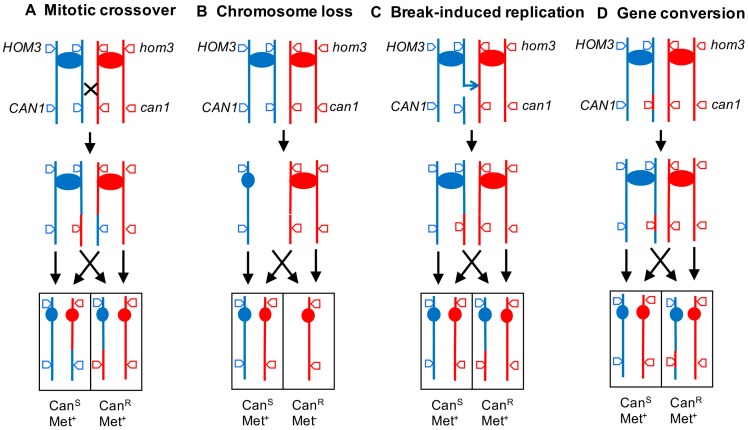
Mechanisms leading to loss of heterozygosity (LOH) in a diploid that is heterozygous for a *can1* mutation. A commonly used assay in yeast to detect LOH involves a diploid that is heterozygous for *can1* and *hom3* mutations located on chromosome V. Strains that are heterozygous for the *can1* mutation are sensitive to canavanine, and strains heterozygous for the *hom3* mutation can grow in medium lacking methionine. The different line colors represent the two homologs, and the ovals show the centromeres. The events are depicted as occurring in cells after replication. (**A**) Mitotic crossover. A crossover between the *can1* marker and the centromere can result in one cell that is homozygous of the *can1* allele and another cell homozygous for the wild-type *CAN1* allele. Both strains remain heterozygous for the *hom3* allele. In the figure, we show the chromosome segregation pattern that results in LOH (indicated by four arrows). An equally frequent segregation pattern in which the recombinant chromatids segregate together will not result in LOH. (**B**) Chromosome loss. Loss of one of the blue chromatids results in one Can^R^ product that is also Met^-^; the other product is identical to the original diploid. (**C**) Break-induced replication. In this mechanism, one blue chromatid is broken, and the acentric fragment is lost. The centromere-containing broken chromatid invades one of the red chromatids, copying its sequences from the point of invasion to the end. The Can^R^ cell has the same phenotype as that produced by a crossover, but the event is non-reciprocal, and the other product is identical to the original diploid. (**D**) Gene conversion. A break in the blue chromatid is repaired using an internal segment of the red chromatid. This mechanism results in an interstitial LOH region.

In addition to methods developed to monitor small changes in the genome, there are a variety of selective and non-selective methods to examine larger changes: large (>1 kb) deletions/duplications, translocations, ploidy alterations, as well as mitotic exchanges between homologs. Although we will limit extensive discussion of such methods to those employed in our own labs, we will briefly mention two widely used selective assays. The first is an assay employed in diploid cells to detect mitotic crossovers and mitotic chromosome loss on chromosome V. For this assay [21], one homolog has the wild-type alleles of *CAN1* and *HOM3*, whereas the other homolog has the mutant alleles. Loss of the wild-type *CAN1* allele by a mitotic crossover (Figure 1A) or by chromosome loss (Figure 1B) results in a canavanine-resistant derivative. Isolates with chromosome loss, unlike those with a mitotic crossover, will be methionine auxotrophs, since *HOM3* encodes an enzyme required to synthesize methionine. This assay allows one to accurately measure the rate of mitotic crossovers between *CAN1* and *CEN5* (a region of about 120 kb), as well the rate of loss of chromosome V.

Although most long terminal loss of heterozygosity (LOH) events in wild-type cells are a consequence of mitotic crossovers, such events can also be produced by a non-reciprocal type of recombination called “break-induced replication” or BIR [22]. In this mechanism (Figure 1C), following a break on one homolog, one chromosome fragment is lost. The centromere-containing fragment invades the intact homolog and replicates the chromosome from the point of invasion to the telomere. The phenotype of the Can^R^ product is identical to that obtained as the result of a crossover. However, if the daughter cell formed at the same time as the canavanine-resistant daughter can be analyzed, a distinction between a crossover and a BIR event can be made, since the daughter formed as a result of the crossover would be *CAN1*/*CAN1*, and that formed as the result of a BIR event would be *can1*/*CAN1*.

In addition to mitotic crossovers, mitotic gene conversions unassociated with crossovers can result in an interstitial region of LOH and a canavanine-resistant derivative (Figure 1D). In mitosis, conversions result in an LOH region with a median size of about 12 kb [23], although conversions as large as 50 kb are sometimes observed [24].

Another selective approach to examine deletion formation and various types of chromosome rearrangements (collectively termed gross chromosomal rearrangements or GCR) has been widely employed by the Kolodner lab and others [25]. In one version of this assay, the *URA3* gene, normally located on chromosome V between *CAN1* and the centromere, is relocated to chromosome V centromere-distal to *CAN1*. By selecting for isolates that become simultaneously resistant to both canavanine and 5-fluoro-orotate, derivatives that have lost the left end of V can be isolated. There are no essential genes between *CAN1* and the telomere. Consequently, most of the isolates have a breakpoint between *CAN1* and the first essential gene located centromere-proximal to *CAN1* (*PCM1*). By PCR analysis and a variety of other approaches, derivatives identified in the GCR assay have undergone a variety of alterations, including deletions coupled with telomere additions, translocations, and inversions.

### 2.2. Analysis of Mitotic Crossovers in Yeast Using Microarrays

Most of the methods described above are restricted to a gene or a single chromosome arm. In this section, we describe the use of microarrays to detect genomic alterations throughout the genome at high resolution [26]. We will first discuss the mapping of mitotic crossovers between homologs in diploid yeast strains by identifying regions of LOH.

What is the rationale for mapping LOH events? Since many (and, perhaps, most) mitotic crossovers are induced by DSBs [22], the breakpoint of the LOH event (the transition between heterozygous markers and homozygous markers) identifies the position of the recombinogenic DNA lesion. Thus, the mapping of many such events in strains under replication stress allows inferences about the nature of fragile sites in yeast.

To map LOH events at high resolution, one needs diploid strains that are heterozygous for many single-nucleotide polymorphisms (SNPs) distributed throughout the genome. In our experiments, the two haploid strains used to construct the diploid (W303-1A and YJM789) were heterozygous for about 50,000 SNPs. Based on previous studies [27], we designed microarrays allowing us to detect LOH for about 13,000 of these SNPs [26]. In our arrays, each of the 13,000 SNPs is represented by four 25-base oligonucleotides, two representing the Watson and Crick sequences of one of the alleles and two representing the Watson and Crick sequences of the other allele. The base representing the SNP is located near the middle of each oligonucleotide.

Genomic DNA isolated from strains that are heterozygous for SNPs at a given position, hybridize about equally well to all four nucleotides. Genomic DNA isolated from strains that are homozygous for the W303-1A-derived SNP at a specific position will hybridize relatively better to the W303-1A-derived oligonucleotides than the YJM789-derived oligonucleotides, because a single mismatch is often sufficient to destabilize short duplexes; similarly, DNA isolated from a diploid that is homozygous for the YJM789-derived SNP at a given position will hybridize better to the YJM789-derived oligonucleotides. The sequences of the oligonucleotides in the arrays and the array designs for our studies are on the Gene Expression Omnibus (GEO) website at the platforms GPL20144 (whole-genome array) and GPL21552 (chromosome IV-specific array).

**Figure 2:**
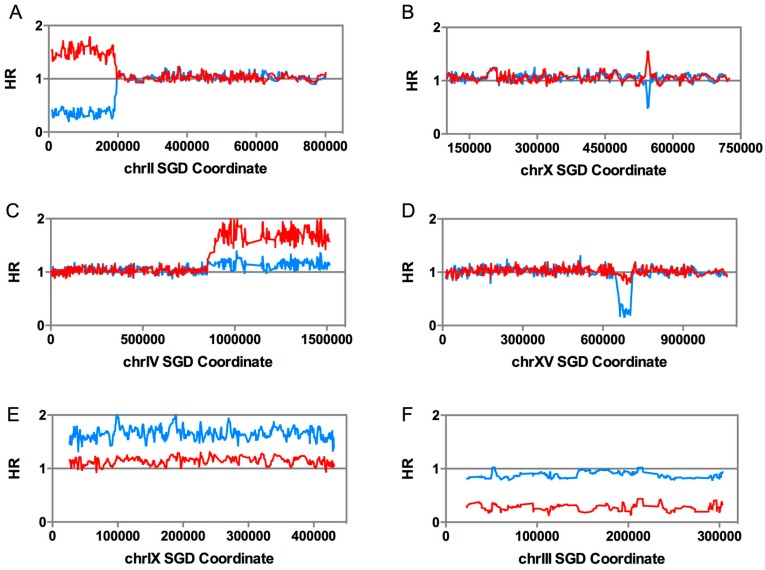
LOH events, large duplications/deletions, and ploidy alterations as detected by single-nucleotide polymorphism (SNP) microarrays. As described in the text, SNP-specific microarrays can be used to detect LOH events, as well as other types of chromosome alterations. In each panel, we show the level of hybridization of W303-1A-specific SNPs (red lines) and YJM789-specific SNPs (blue lines). These values are normalized to the level of hybridization observed in a heterozygous diploid. A hybridization ratio (HR) of 1 indicates heterozygosity, and values about 1.5 or 0.2 indicate 2-fold increases or decreases in hybridization of the SNPs, respectively. (**A**) Terminal LOH event. Such an event could be caused by either a crossover or break-induced replication (BIR) event. (**B**) Interstitial LOH event. These events reflect gene conversions. (**C**) Terminal duplication. A segment of the chromosome is duplicated. As described in the text, such events are usually observed in cells that also have a terminal deletion, and likely reflect a double-stranded DNA break (DSB) on one chromosome that is repaired by a BIR event involving a different homolog, resulting in a translocation. (**D**) Interstitial deletion. In this event, there was a deletion on the blue homolog. Such deletions usually involve homologous recombination between non-allelic repeats. (**E**) Trisomy. In this cell, the blue homolog is present in two copies, and the red homolog in one copy. (**F**) Monosomy. In this cell, the red homolog was lost, and the blue homolog retained. SGD: *Saccharomyces* Genome Database.

The whole-genome microarrays allowed us to map mitotic crossovers and other chromosome alterations throughout the genome to a resolution of about 1 kb. An example of the analysis of a recombination event on chromosome II is shown in Figure 2A. In this figure, the level of hybridization to the W303-1A-derived SNPs (normalized to the level of hybridization in the heterozygous diploid) is shown in red, and the level of hybridization to the YJM789-specific SNPs is shown in blue. The strain is heterozygous for SNPs between the right telomere and *Saccharomyces* Genome Database (SGD) coordinate 195 kb and, then, becomes homozygous for W303-1A-derived SNPs from coordinate 195 kb to the left telomere. An interstitial LOH event (gene conversion) is shown in Figure 2B; in this example, sequences from the W303-1A-derived homolog located between SGD coordinates 542 kb and 549 kb are duplicated. Note that, for both Figure 2A,B, the increase in hybridization for the SNPs derived from one homolog is balanced by the decrease in hybridization for SNPs derived from the other homolog.

**Figure 3:**
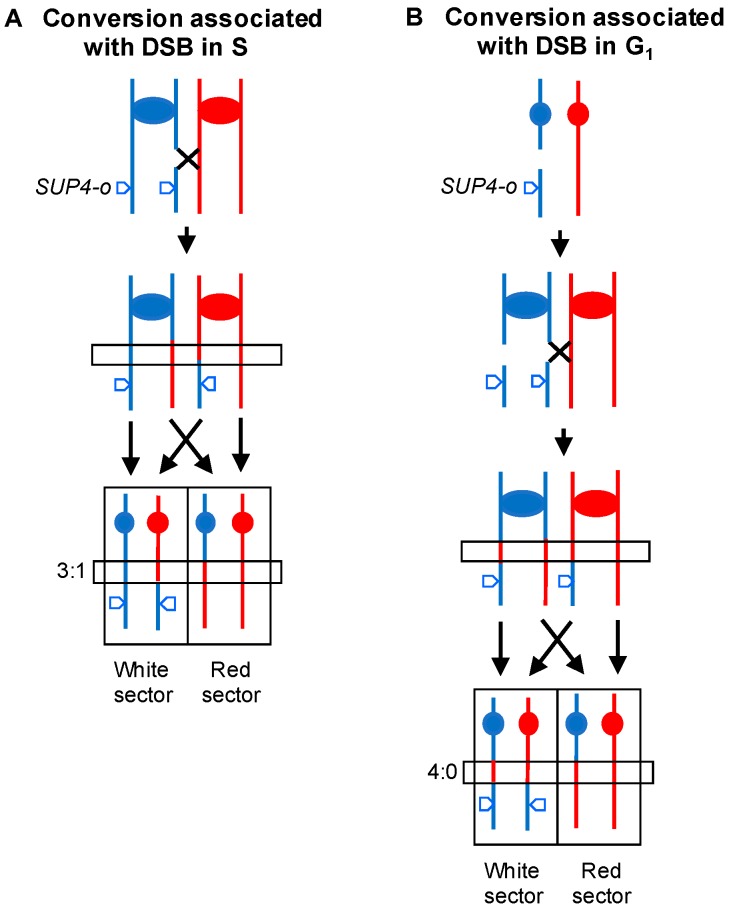
Detecting gene conversion events associated with crossovers initiated in G_2_ or G_1_. A diploid is constructed that is heterozygous for an insertion of the *SUP4-o* ochre suppressing tRNA gene, and homozygous for the *ade2-1* ochre-suppressible allele. The diploid forms pink colonies, but derivatives that have zero or two copies of *SUP4-o* result in red or white colonies, respectively. Thus, crossovers can produce a red/white sectored colony. These sectors are analyzed by SNP arrays to determine the location of the recombination breakpoints. The region of conversion (boxed in the figure) is detected as a difference in the location of the breakpoints in the two sectors. (**A**) Crossover as a consequence of a DSB on one chromatid. As a consequence of the conversion associated with repair of a single broken chromatid, a 3:1 conversion (a region in which 3 of the chromatids contain sequences from the red homolog and one contains sequences from the blue homolog) is observed. (**B**) Crossover reflecting the repair of two DSBs. We show a broken chromosome that is replicated to yield two chromatids broken at the same position. The conversion tracts associated with these repair events result in a 4:0 region. To explain the pattern of LOH, we suggest that one break is repaired to generate a crossover, and the second is repaired to produce a conversion event unassociated with a crossover.

In some of our studies, we examined the recombinant products found in both daughter cells involved in the recombination event. For most of these studies, we used the system shown in Figure 3 [23]. The hybrid diploid is homozygous for the *ade2-1* allele (an ochre mutation), and heterozygous for the *SUP4-o* gene (a tyrosine-inserting tRNA gene that suppresses ochre mutations). The *SUP4-o* gene is located near the right telomere of chromosome IV. In diploids homozygous for *ade2-1*, derivatives that have zero, one, or two copies of *SUP4-o* form red, pink, and white colonies, respectively. Thus, a crossover between the centromere of IV and *SUP4-o* (a distance of 1 Mb) can produce a red/white sectored colony, whereas the starting strain forms pink colonies. A BIR event can produce either white/pink or red/pink sectored colonies.

Cells purified from the red and white sides of sectored colonies can be examined separately by microarray analysis to determine important features of the recombination event. From many previous studies of both meiotic and mitotic recombination in yeast, it has been shown that some conversion events occur without an associated crossover, whereas others occur in association with crossovers [22,28]. In the current models of recombination, both types of events are initiated by double-stranded DNA breaks, followed by heteroduplex formation between the broken molecule and an intact template.

In the system shown in Figure 3, most (87%) spontaneous crossovers are associated with gene conversion events [23]. Conversions are detected by observing different breakpoints for the LOH events in each sector. In Figure 3A, in the boxed region, three of the four chromosomes that were involved in the recombination event have sequences derived from the W303-1A homolog, and one has YJM789-derived sequences; this type of conversion is called a “3:1” event. Very surprisingly, about two-thirds of the spontaneous crossovers were associated with a different type of conversion (4:0), in which all four chromatids had sequences derived from one of the two homologs (Figure 3B) [23,29]. Our interpretation of this pattern is that most of the spontaneous lesions that produced recombination between the homologs are generated in unreplicated chromosomes, likely in G_1_ of the cell cycle. Following DNA replication, the resulting broken chromosomes are repaired in G_2_ to produce the recombinant products. Thus, the analysis of events in sectored colonies allows us to make conclusions about the timing of the recombinogenic DNA lesions. In addition, the analysis of sectored colonies allows one to determine whether terminal LOH events are a consequence of crossovers or BIR. In our studies of genomic rearrangements induced by replication stress, we examined both unselected colonies as well as sectored colonies.

### 2.3. Diagnosis of Other Genomic Alterations Using Microarrays

In addition to recombination between homologs, the microarrays allow diagnosis of a number of other types of large chromosome alterations, including terminal deletions/duplications (duplication shown in Figure 2C), interstitial deletions/duplications (deletion shown in Figure 2D), trisomy (Figure 2E), and monosomy (Figure 2F). In all of these alterations, unlike LOH events, there is a net loss or gain of sequences. In addition to those events shown in Figure 2, uniparental disomy (UPD; one homolog is lost, and the other is duplicated) can be detected.

### 2.4. Systems for Inducing DNA Replication Stress by Depleting DNA Polymerases

**Figure 4:**
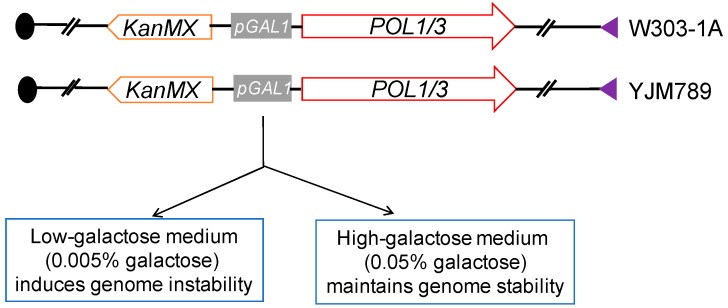
Yeast strains used to analyze the effects of low levels of DNA polymerase on genome stability. Two types of strains were constructed, one homozygous for a *GAL-POL1* gene (a galactose-inducible promoter fused to the coding sequence of *POL1*, encoding the catalytic subunit of Polα) and one homozygous for *GAL-POL3* (the *GAL* promoter fused to the gene encoding the catalytic subunit of Polδ). When these strains are grown in low levels of galactose, the amount of the relevant DNA polymerases is reduced by about a factor of 10, resulting in genomic instability. Cells grown in high-galactose medium have relatively stable genomes.

In mammalian studies of chromosome breaks induced by replication stress, the cells are usually treated with drugs that inhibit DNA polymerase (aphidicolin) or that reduce pools of nucleotides [7]. In our experiments, we applied a more specific type of replication stress; we depleted the levels of either Polα or Polδ using an inducible promoter [12,13,30]. The native promoters of genes *POL1* (encoding the catalytic subunit of Polα) or *POL3* (encoding the catalytic subunit of Polδ) were replaced by the galactose-inducible *GAL1* promoter (Figure 4). In these strains, when grown in low-galactose medium (0.005% galactose, 3% raffinose), the expression levels of the DNA polymerases were reduced by approximately 10-fold compared to the levels in wild-type cells [30,31]. This reduction results in slower growth rates (reflecting an extended S-phase), sensitivity to several DNA damaging agents, and greatly elevated rates of genomic alterations [30,31,32]. These effects were substantially reduced by growing the strains in high-galactose medium (0.05% galactose, 3% raffinose); this growth condition resulted in a level of DNA polymerase expression that was about 3-fold higher than in wild-type cells.

Our initial experiments analyzing the genome-destabilizing effects of low levels of DNA polymerases were done by examining illegitimate mating of a *MAT*α haploid with the *GAL-POL1* construct with a wild-type *MAT*α haploid [30]. Strathern et al. [33] had shown that loss, or inactivation, of the *MAT*α gene located on the left arm of chromosome III allows *MAT*α haploids to mate with other *MAT*α haploids; normally, mating in yeast occurs only between haploids with different mating types. Analysis of the resulting diploids showed that low levels of Polα greatly (100-fold) elevated the rates of loss of chromosome III, as well as deletions of the right arm of III [30]. Most of the deletions were the result of non-reciprocal translocations between a pair of transposable elements (Ty elements) located centromere-proximal to the *MAT* locus with Ty elements located on other chromosomes.

Although these illegitimate mating experiments resulted in useful information about the nature of genome instability in strains under replication stress, we performed most of our subsequent analysis in diploid strains (described above) that were heterozygous for many SNPs, since this type of diploid allowed detection of LOH events throughout the genome. The results of such studies are described below.

## 3. Genomic Instability in Wild-Type Strains and in Strains with Low Levels of Replicative DNA Polymerases

Although depletion of either Polα or Polδ results in high levels of genomic instability, the types of alterations are quantitatively different. Therefore, the results obtained with these two types of replication stress will be discussed separately. Before discussing these experiments, we will first describe the level of genetic instability observed in wild-type strains.

### 3.1. Genetic Instability in Wild-Type Diploids

As expected, in the wild-type diploid generated by crossing the haploids W303-1A and YJM789 (henceforth referred to as the “hybrid diploid”), LOH events were found at a very low level. Using SNP arrays, in analyzing 10 strains subcultured for 250 cell divisions, we found only one terminal LOH event (similar to that shown in Figure 2A) and four interstitial LOH events (similar to that shown in Figure 2B), a frequency of about 4 × 10^−4^/cell division, and 1.6 × 10^−3^/division for terminal and interstitial LOH events, respectively [34]. Using an assay specific for the detection of LOH events on the right arm of chromosome IV, we measured a rate of about 3 × 10^−5^/division [23]. Since the right arm of IV contains about 8% of the genome, we can extrapolate this rate to 3.8 × 10^−4^/division/genome, similar to the rate estimated by the whole-genome analysis.

**Figure 5:**
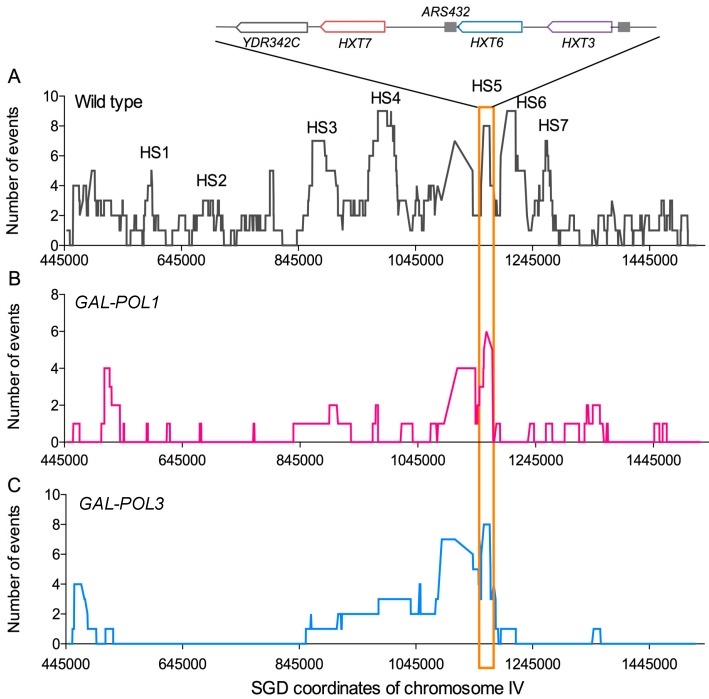
Mitotic recombination hotspots on the right arm of chromosome IV in wild-type, *GAL1-POL1*, and *GAL1-POL3* strains. Crossover events on the right arm of chromosome IV were selected using the system described in Figure 3, and events were mapped using SNP microarrays. The Y axis of the plots represent the number of times a SNP is included in conversion tracts associated with a crossover; the X axis shows the SGD coordinates from *CEN4* to the right telomere. (**A**) Recombination events in the wild-type diploid [23]. Hotspots of recombination activity are labeled HS1–HS7. HS5, which contains three directly oriented *HXT* genes, has high levels of recombination in all three strains. (**B**) Recombination in cells with low levels of Polα [12]. (**C**) Recombination in cells with low levels of Polδ [13].

From the mapping of crossovers on chromosome IV, we identified a number of recombination hotspots (Figure 5A). Two (HS3 and HS4) were associated with inverted pairs of Ty elements, and a third (HS5) contained three directly repeated *HXT* genes; the HS3 and HS4 hotspots are G_1_-specific [23]. The sequences at the breakpoints of crossovers were examined for overrepresentations of various chromosome elements (replication origins, tRNA genes, transposable elements, etc.). Of approximately twenty associations examined, spontaneous recombination events were non-randomly associated with Ty elements and delta repeats (the long-terminal repeats (LTRs) associated with Ty elements), tRNA genes, G4 (quadruplex) motifs, replication termination regions, and pause sites for the Rrm3 helicase. Most of these sequences are associated with chromosome regions in which the DNA forks move slowly or exhibit a transient stall (reviewed by Azvolinsky et al. [35]). As described in the Introduction, in human cells, fragile sites are also associated with late-replicating regions and/or stalled replication forks [7]. In addition, as in yeast, fragile sites often contain sequences that have the potential to form secondary structures. However, a subset of fragile sites in humans contain AT-rich microsatellites [7], an association that is not found in yeast. Thus, certain features of fragile sites appear conserved between yeast and mammalian cells, whereas others are organism-specific.

In addition to the low rate of mitotic recombination events, the wild-type diploid had very low rates of large deletions/duplications and ploidy alterations. In the sample of ten strains subcultured 20 times, we observed no large (>5 kb) deletions or duplications, and only one event of trisomy [34]. Among 145 wild-type diploid isolates that underwent ~2000 cell divisions, Zhu et al. [20] identified 29 trisomy and 2 monosomy events. From the Zhu et al. data, the rates of gain and loss of whole chromosomes were 9.7 × 10^−5^ and 0.7 × 10^−5^ events per diploid genome per generation, respectively. Since most aneuploid strains grow more slowly than wild-type strains [36], these rates may be underestimates. In addition, since it is possible that trisomic strains have less of a growth disadvantage than monosomic strains, the ratio of trisomy to monosomy events may be skewed.

In addition to whole-chromosome changes that produce aneuploids, wild-type diploids have low rates of uniparental disomy (described above). The rate of UPD for chromosome V was 10^−7^/cell division, about 10-fold lower than the rate of mitotic recombination for the same chromosome [37].

The yeast *S. cerevisiae* has two arrays of tandemly repeated genes, the ribosomal RNA genes (9 kb repeats duplicated 75–150 times on chromosome XII; [38]) and the *CUP1* genes (a repeat of 1 to 2 kb found in about 10 copies in most isolates on chromosome VIII; [39]). The rates of crossovers between homologs within these arrays are 1.9 × 10^−3^/division for the rRNA genes [40] and 6 × 10^−6^/division for the *CUP1* array [41].

By measuring the loss of markers inserted within the rRNA or *CUP1* arrays, the rates of intrachromatid/sister-chromatid recombination events have also been examined. These rates are about 1.2 × 10^−3^/division [40] and 1.5 × 10^−4^/division [41], for the rRNA genes and the *CUP1* array, respectively. These rates are minimal estimates of the rates of intrachromatid/sister-chromatid events per tandem array, since only those events that delete the marker are detected. However, in a study in which alterations in the length of the *CUP1* cluster were measured by gel electrophoresis, the rate of alterations (presumably generated by intrachromatid/sister-chromatid recombination) was 3.4 × 10^−4^/division [42], similar to the rate calculated by marker loss. 

Lastly, based on whole-genome sequence analysis in wild-type strains not isogenic to those used in our study, the rate of single-base substitutions and small (<700 base) in/dels is also very low. Zhu et al. [20] found rates of 1.7 × 10^−10^ substitutions/base/cell division and 5 × 10^−12^ in-dels/base/cell division in a diploid strain, and Nishant et al. [19] observed roughly similar values in a wild-type diploid. The stability of the genome in wild-type yeast strains is in dramatic contrast to the instability observed in strains with low levels of DNA polymerase.

### 3.2. Genetic Instability in Strains with Low Levels of Polα

Studies of the effects of low levels of Polα in yeast are in five papers: [12,30,40,43,44]; the discussion below emphasizes the paper by Song et al. [12], since this study was the only one to include a genome-wide analysis.

#### 3.2.1. Loss of Heterozygosity Events Resulting from Mitotic Recombination between Homologs

In hybrid diploid strains, two types of LOH analysis were done: non-selective whole-genome studies [12] and studies of events selected to occur on one chromosome arm [12,44]. For the non-selective studies, we allowed the hybrid strain with the *GAL-POL1* construct to grow from a single cell to a colony (representing about 25 cell divisions) on low-galactose medium. From 25 independent colonies, we then repurified isolates on high-galactose medium, and examined them for LOH using SNP-specific microarrays. Among the 25 isolates, there were 201 mitotic recombination events. One hundred and fifteen of these events were terminal LOH events that could reflect either mitotic crossovers or BIR events, and 86 were interstitial LOH events likely to represent gene conversions unassociated with crossovers [12]. Compared to the frequencies measured in wild-type strains, terminal LOH events were elevated about 450-fold, and gene conversions were elevated about 90-fold.

Both terminal and interstitial LOH events were distributed widely throughout the genome with no very strong hotspots [12]. As described previously, the terminal LOH events in unsectored colonies could represent either a mitotic crossover or a BIR event. For all of the terminal and interstitial LOH events, we determined a “window” (usually about 20 kb in size) that contained the transition between heterozygous and homozygous SNPs. These breakpoints should contain the site of the initiating recombinogenic DSB. The following chromosome elements/sequence motifs were overrepresented at the breakpoints: non-coding RNA genes, solo deltas, G4 sequences, binding sites for Rrm3p, and replication-termination sequences. All of these categories, except non-coding RNA genes, were previously observed to be overrepresented for spontaneous events. The simplest explanation of these findings is that regions that are slow to replicate are often hotspots for DNA breaks, even in wild-type strains under normal growth conditions. Under replication stress, these same regions break at elevated frequencies.

Mitotic crossovers in cells with low Polα were also examined in red/white sectored colonies using the system shown in Figure 3. The mapping of these events is shown in Figure 5B. Most of the hotspots observed in cells with wild-type levels of DNA polymerase were not evident in the cells with low Polα, although HS5 associated with the *HXT* genes was still present. In a similar analysis of sectored colonies using a *GAL-POL1* strain with markers on chromosome III, Rosen et al. [44] found a strong hotspot for recombination associated with an inverted pair of Ty elements, previously referred to as FS2 [30]. It is puzzling that FS2 is a hotspot on III under conditions of low Polα, whereas the two pairs of inverted Ty elements on chromosome IV are hotspots for spontaneous events, but not under conditions of low Polα. One possibility is that the DSBs occurring in the Ty pairs on IV are preferentially repaired by sister chromatid exchange (which would not lead to LOH) relative to the pair on chromosome III, although other explanations are also possible.

The other important conclusion from the analysis of sectored colonies was that all of the crossovers (29 of 29) were associated with 3:1 conversions, rather than 4:0 conversions [12]. In a study of crossovers on chromosome III, Rosen et al. [44] found that >90% of the conversion events had the 3:1 conversion pattern. These results demonstrate that most of the recombinogenic DNA lesions in strains with low Polα are generated in S or G_2_, consistent with the possibility that this condition results in an elevated rate of broken replication forks.

#### 3.2.2. Large (>5 kb) Deletions and Duplications

Among 25 *GAL-POL1* isolates grown in low-galactose medium, we observed 43 interstitial in/dels (8 duplications and 35 deletions), and 34 terminal in/dels (16 duplications and 18 deletions) [12]. The frequencies of interstitial and terminal in/dels per strain per cell division are about 7 × 10^−2^ and 5 × 10^−2^, respectively. It is difficult to determine a frequency of these events relative to wild-type, since we observed no large in/dels in wild-type diploids of the same genetic background, and Nishant et al. [19] found no such alterations in a different diploid background (20 isolates that had divided 1740 times). If we assume that there was a single large in/del in the ten isolates subcultured 250 times in our previous experiment [34], we calculate that the frequency of such events relative to the wild-type strain is elevated at least 300-fold by low levels of Polα.

**Figure 6:**
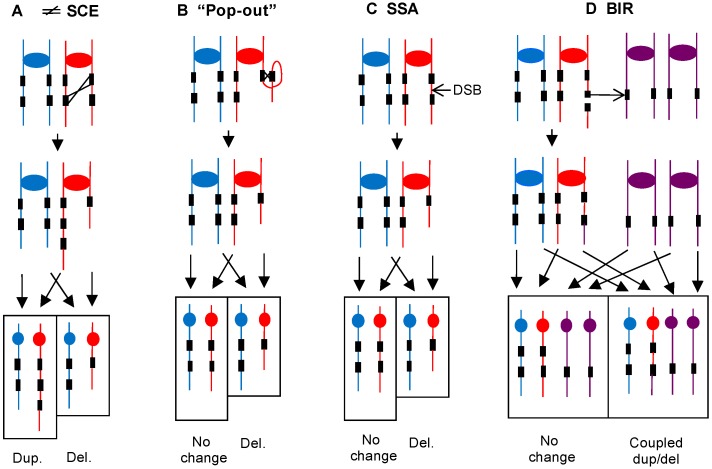
Homologous recombination between non-allelic repeats as a mechanism for generating large deletions and duplications. Blue and red lines show the two homologs of the diploid, and the purple lines indicate a different homolog. Black rectangles depict directly repeated genes. Arrows near the bottom of each panel show patterns of segregation with rectangles outlining the two daughter cells. Duplicated and unduplicated centromeres are shown as ovals and circles, respectively. (**A**) Unequal sister-chromatid exchange. Unequal crossing over between non-allelic repeats will produce one chromosome with a deletion and one with a duplication. Such unequal crossovers could also occur between homologs. (**B**) “Pop-out” event. An intrachromatid crossover between direct repeats will produce a chromosome with a deletion and a circular DNA molecule. The circle would likely be lost because it lacks a centromere. (**C**) Single-strand annealing (SSA). In this mechanism, a DSB occurs between two directly oriented repeats. Following processing of the broken ends, the repeats can anneal, resulting in loss of one repeat and the intervening DNA sequences [22]. (**D**) BIR. A DSB occurs in a repeat in one of the red chromatids with loss of the terminal fragment. The centromere-containing broken end invades a repeat on a non-homolog (purple chromatids) and copies the sequences from the point of invasion to the chromosome terminus. The resulting event generates a translocation. The daughter cell with the translocation will contain a large terminal deletion of “red” sequences and a large terminal duplication of “purple” sequences.

Large interstitial deletions and duplications are likely produced by different mechanisms than those that result in large terminal deletions and duplications. One common source of large interstitial deletions in yeast is homologous recombination between repeated genes located at non-allelic positions on either the sister-chromatid or on the homologs. Such exchanges can produce either deletions or duplications (Figure 6A), as well as other rearrangements to be described below. Interstitial deletions can also be generated by intrachromatid crossovers (“pop-outs”, Figure 6B) and single-strand annealing (Figure 6C) (reviewed by Symington et al. [22]). It is possible that all three mechanisms shown in Figure 6A–C are responsible for some proportion of deletions.

All of the 43 interstitial in/dels had repetitive sequences at the deletion/duplication endpoints. The Ty retrotransposons are the largest class of dispersed repeated elements in *S. cerevisiae*, and interstitial deletions often involve such elements (reviewed by Mieczkowski et al. [45]). In strains with low Polα, 21 of the strains had two Ty elements at the breakpoints of the rearrangement, and one had a Ty element at one breakpoint and a solo delta at the other. Two of the duplications were generated by recombination between other classes of repeats (*HXT* genes and the *MAT*/*HMR* loci) [12].

The in/dels shown in Figure 6 occur between repeats that are interspersed with single-copy DNA sequences. In/dels also occur between tandemly repeated genes by the same mechanisms. In *S. cerevisiae*, as described above, the two major families of tandem repeats are the ribosomal DNA and *CUP1* arrays. Using diploids in which the rDNA array was flanked by selectable markers, Casper et al. [40] showed that low Polα elevated the rate of loss of a marker inserted in the rDNA array by 4-fold. Using a PCR-based assay and a yeast strain with the *GAL-POL1* construct, Salim et al. [46] showed that low levels of DNA polymerase α, as well as a number of other DNA-stress-inducing mutants, had rDNA arrays that were shorter than the starting strain. This reduction in array size was observed in 3 of 3 isolates subcultured for 25 cell divisions in low-galactose medium. They also showed that cells with shorter arrays had a growth advantage under conditions of replication stress. Although there are a number of interpretations of why strains with short arrays have a selective growth advantage [46], one possibility is that the replication origins in the rDNA (one origin/repeat) compete for replication factors with non-rDNA origins, and that a reduction in the number of the rDNA origins allows more efficient replication of the other origins under conditions of replication stress [47,48]. The *CUP1* arrays were also unstable in strains with low Polα. Nineteen of 25 isolates that underwent 25 cells divisions had alterations, with deletions exceeding duplications 17 to 2 [12]. This observed frequency of alterations is 84-fold higher than observed in wild-type strains [42].

In addition to interstitial in/dels, strains with low Polα had elevated levels of terminal deletions and duplications. In many of the isolates, these terminal alterations were coupled with a terminal duplication in the same isolate. In addition, most of the coupled in/dels had Ty elements or other repeats at the in/del breakpoints. Of 34 terminal in/dels, 32 had Ty, delta, or *HXT* repeats at their breakpoints [12]. Events that shared these properties were detected previously in low Polα strains selected to have a recombination event on chromosome III [30], and in the genetically unstable *tel1 mec1* strain [49].

One likely mechanism for generating coupled terminal in/dels is shown in Figure 6D. A break occurs within a repeat, followed by loss of the acentric fragment. The centromere-containing fragment invades a non-allelic repeat on another homolog, and the resulting BIR event could produce a coupled deletion and duplication. Alternatively, a reciprocal crossover between repeats on non-homologous chromosomes associated with a particular pattern of segregation could produce both a deletion and duplication within one daughter cell. The net result of either of these mechanisms is a translocation, and a chromosome of the expected size that hybridized to probes derived from different homologs was detected by contour-clamped homogeneous electric field (CHEF) gel electrophoresis in two studies [30,49]. Based on the frequency of the coupled deletion/duplications, the approximate rate of these translocation-forming mechanisms is about 2 × 10^−2^/isolate/cell division in strains with low Polα [12].

In summary, most of the large in/dels observed in the low Polα diploids are a consequence of homologous recombination between non-allelic repeats. Two further points should be mentioned. First, it is possible that the types of large in/dels would be more varied in haploid strains than in diploids, since deletions and duplications could be formed by non-homologous end-joining (NHEJ) events in haploids; this pathway is turned off in *MAT***a**/*MAT*α diploids [22]. In a *tel1 mec1* strain in which NHEJ was active, one translocation involved NHEJ [49]. Even in haploid cells, however, the preferred pathway for generating large in/dels is homologous recombination [50]. Second, in mammalian cells, the preferred method of DSB repair is NHEJ [51], although homologous recombination between non-allelic repeats is an important source of copy-number variation [52].

#### 3.2.3. Rate of Aneuploidy

Among the 25 unselected isolates in the low Polα strain, we found 55 monosomies and 12 trisomies. The observed frequencies per strain per cell division (8.8 × 10^−2^ for monosomies and 1.9 × 10^−2^ for trisomies) are about 1.3 × 10^4^-fold and 200-fold elevated, relative to those in the wild-type strain, respectively. The monosomy/trisomy ratio in the *GAL-POL1* strain (4.6) is very different from that observed in the wild-type strain (0.07; [20]), perhaps a consequence of selection against monosomic strains in long-term subculturing experiments as discussed above. Although there was non-randomness in the recovery of monosomes, with 32 of the 55 monosomes resulting from the loss of chromosomes V, VI, or XIII, there was no simple relationship between chromosome size and monosomy [12].

The high frequency of aneuploidy in strains with low levels of DNA polymerase α could have two different causes. First, low levels of Polα could result in inefficient DSB repair by homologous recombination. Failure to repair a broken chromosome would lead to monosomy. Alternatively, slow DNA synthesis could lead to segregation of an incompletely replicated chromosome. This type of event could produce one monosomic daughter cell and one trisomic daughter.

In addition to monosomic and trisomic chromosomes, we observed 12 UPD events. In strains with UPD events, there are two copies of the homolog, but both are derived from one of the haploid parents. UPD events are often explained as the consequence of a chromosome loss, followed by a second non-disjunction event that duplicates the remaining homolog. Although this mechanism is plausible, at least some UPD events are a consequence of a cell division, in which one cell receives two copies of one parental homolog and the other cell gets two copies of the other homolog [37].

#### 3.2.4. Rate of Base Substitutions and Small Insertions/Deletions

In contrast to the hypermutable phenotype of strains with low levels of Polδ, low levels of Polα elevated mutation rates at the *CAN1* locus by only 2-fold [30]. This modest elevation was, however, statistically significant.

### 3.3. Genetic Instability in Strains with Low Levels of Polδ

The publications relevant to these studies are [13,31,32]. In the most comprehensive of these studies, we used a hybrid diploid that was isogenic with that used for Polα analysis, except the *GAL-POL1* construct was replaced with *GAL-POL3* [13]. In this study, isolates were grown from a single cell to a colony in low-galactose medium, and 35 independent isolates were examined by SNP microarrays. Fifteen of these isolates were also examined by whole-genome sequencing.

#### 3.3.1. Loss of Heterozygosity Events Resulting from Mitotic Recombination between Homologs

By microarray analysis of 35 unsectored colonies grown in low-galactose medium, we detected 21 interstitial events (gene conversions; frequency 15-fold higher than wild-type) and 69 terminal events (crossovers or BIR, frequency 200-fold higher than wild-type). As observed for the low Polα strains, the LOH events were broadly distributed throughout the genome with no very strong hotspots. The analysis of chromosomal elements at the breakpoints of the LOH events indicated a significant overrepresentation of G4 quadruplex sequences, Ty elements, autonomously replicating sequences (ARS elements), and gamma-H2AX-enriched regions; gamma-H2AX-enriched regions have been shown to overlap with loci prone to replication-fork stalling [53]. Thus, similar to the motifs identified in the low Polα, recombinogenic DNA lesions in the low Polδ strains tend to be located in regions of slow-moving or stalled replication forks. Not all of the motifs identified in the low Polα strains were statistically significant in the low Polδ strains. In part, this discrepancy may simply reflect the smaller number of events analyzed. When the ratios of observed to expected associations were compared for all tested chromosomal elements for the low Polα and Polδ strains, these values correlated with a coefficient of 0.75 with a *p* value of 0.002 [13].

In addition to the microarray analysis, we examined 15 of the 35 unsectored colonies by whole-genome sequencing. All of the events detected by microarrays were confirmed by sequencing. Although no new terminal LOH events were detected by sequencing, we found additional gene conversion events. Sixteen “new” conversion events were detected among the 15 isolates sequenced versus the 6 events detected by microarrays. The sequencing-specific events had a much shorter median length (1.5 kb) than observed by microarrays (7.1 kb). It is possible that low Polδ results in a novel type of conversion. In a study in which we examined UV-induced conversions by both SNP arrays and DNA sequencing, we found little difference in the number detected, 23 by microarrays and 26 by DNA sequencing [26].

The pattern of crossovers and associated conversions on the right arm of IV was also examined by the sectored-colony approach. The frequency of sectored colonies was 1.4 × 10^−2^, representing an elevation of about 500-fold relative to the wild-type strain [13]. The positions of crossovers in the 34 sectored colonies examined are shown in Figure 5C. The pattern in the low Polδ is similar to that observed in the low Polα strain. In both of these strains, HS5 (containing the directly repeated *HXT* genes) is prominent, and both strains lack the HS3 and HS4 hotspots seen for spontaneous recombination events (Figure 5A). In addition to acting as a hotspot for recombination between homologs, the *HXT* genes at this position were preferred sites for deletions and duplications, as described below.

Based on the analysis of sectored colonies, we also concluded that the DNA lesions in strains with low Polδ were likely formed in S or G_2_. Of the 19 simple conversions that were associated with crossovers, all were 3:1 events, rather than 4:0 events [13]. We infer that most of the recombinogenic lesions were likely associated with broken replication forks.

#### 3.3.2. Large (>5 kb) Deletions and Duplications

We observed 41 large (>5 kb) interstitial in/dels (37 deletions and 4 duplications) [13], a frequency of about 4.7 × 10^−2^/division/isolate. This frequency is at least 100-fold greater than observed in the wild-type diploid [34]. Of these 41 large changes, all involved direct repeats. Ten had non-allelic Ty elements at the breakpoints, four had solo delta elements at the breakpoints, and six occurred between three closely linked *HXT* genes on chromosome IV [13]. 21 in/dels were observed in the *CUP1* array, 20 deletions, and one duplication; this frequency is about 70-fold elevated relative to that observed in wild-type strains. As discussed above, the involvement of repeats suggests that the duplications are a consequence of non-allelic homologous recombination, and the deletions could reflect non-allelic recombination between sister chromatids or homologs, intrachromatid recombination, or single-strand annealing.

By analyzing the number of “reads” in the genomic sequence performed with 15 isolates, we found frequent deletions within the ribosomal RNA gene cluster [13]. The number of rDNA repeats in the diploid before exposure to low-galactose medium was about 122. Following one cycle of growth in low-galactose medium, 13 of 15 isolates had a reduced number of repeats, with some isolates having about half the starting number. As described previously, Salim et al. [46] showed that low levels of Polα, as well as a number of other DNA-stress-inducing mutants, led to shortened rDNA arrays. These results confirm that a reduction in rDNA is a general response to DNA replication stress.

Sixteen large terminal in/dels were found among 35 *GAL-POL3* isolates grown from a single cell to a colony in low-galactose medium [13]; 15 of 16 breakpoints for these rearrangements were at Ty or delta sequences. Assuming that each coupled in/del is a consequence of a single event, such as shown in Figure 6D, we calculate that the approximate frequency of translocation formation by this mechanism is about 9 × 10^−3^/division/isolate, similar to the frequency found in low Polα strains.

#### 3.3.3. Rate of Aneuploidy

The frequencies of both monosomy and trisomy were substantially elevated in strains with low polymerase δ. The frequencies for monosomes and trisomes (events/strain/cell division) were 1.2 × 10^−1^ (1.6 × 10^4^ greater than wild-type) and 9 × 10^−3^ (95-fold greater than wild-type), respectively; UPD events were observed at a frequency of 1.3 × 10^−3^. Fifty-six percent of the monosomic chromosomes were III, V, and XIV. The two largest chromosomes (IV and XII) were underrepresented as monosomes (2 of 97 monosomes).

Underrepresentation of certain chromosomes as aneuploids could be a consequence of negative growth effects when these chromosomes are aneuploid. Alternatively, certain chromosomes, when aneuploid, may alleviate the slow growth of the *GAL-POL3* strains. Another possibility is that certain chromosomes are preferred substrates for DNA damage and/or incomplete replication under conditions of low Polδ, leading to chromosome loss.

#### 3.3.4. Rate of Base Substitutions and Small Insertions/Deletions

Kokoska et al. [31] showed that the rate of *CAN1* mutations was elevated about 10-fold for *GAL-POL3* strains grown in 0.005% galactose, compared to the same strain grown in 0.05% galactose. In addition, the spectrum of mutations was altered. In strains with low Polδ, more than half of the mutations were small (15–53 bp) deletions, compared to less than 10% observed in wild-type strains. These deletions were flanked by short direct repeats. These results were extended to the whole genome by sequencing of 15 Polδ isolates [13]. This analysis showed that low Polδ elevated single-base substitutions were about 30-fold and small deletions about 500-fold compared to wild-type strain. Since the small deletions were flanked by short (3–9 bp) direct repeats, these observations suggest that low levels of DNA polymerase δ result in elevated frequencies of DNA polymerase slippage [13]. The elevated rate of base substitutions may reflect the recruitment of the error-prone DNA polymerase ζ, since a significantly increased frequency of GC/CG transversions was found in the low-polymerase-δ strains, and this alteration is characteristic of DNA polymerase ζ [54].

#### 3.3.5. Evolution of the Genome under Conditions of Low Polδ

There are two examples of how the certain genomic alterations were selected by replication stress imposed by low Polδ. First, as discussed above, most isolates reduced the amount of rDNA, an alteration that has a growth advantage under multiple conditions of DNA stress [46]. Second, of the 35 isolates allowed to form a colony in low-galactose medium, 4 grew conspicuously better than the starting strain [13]. Three of these 4 isolates had a deletion of a region of the *GAL* promoter that regulated the synthesis of *POL3*. Since all three of these deletions removed the binding site of Mig1p, a negative regulator of the *GAL* promoter, the net effect of these deletions was likely to increase the transcription of *POL3*. We also found that one isolate had a duplication of a region of IV that contained the *GAL-POL3* construct, resulting in a diploid with three copies of *GAL-POL3*. By making a derivative of this strain that had only two copies of the *GAL-POL3* gene, we showed that an additional copy of *GAL-POL3* resulted in a selective growth advantage [13]. In summary, strains with low Polδ initially have a selective growth disadvantage, but evolve quickly to produce fast-growing derivatives.

### 3.4. Comparison of Genomic Instability Induced by Low Levels of Polα or Polδ

The genomic instability induced by low levels of Polα or Polδ share a number of common features (summarized in Table 1). Both conditions substantially (often >100-fold) elevate mitotic recombination between homologs, and the formation of large in/dels and translocations. The large in/dels and translocations usually reflect recombination between Ty elements or other non-allelic repeats. The *CUP1* tandem array is reduced in size in strains with low levels of Polα or Polδ; the rDNA array is also reduced in strains with low Polα or Polδ. These observations could reflect a high rate of single-strand annealing within these clusters (Figure 6C) or other mechanisms (Figure 6A,B), followed by selection for strains with smaller rDNA gene clusters as discussed above.

The terminal and interstitial LOH events in both the low Polα or Polδ strains were widely distributed through the genome. In both types of strains, at the breakpoints of the LOH events, we found an overrepresentation of chromosome elements known to be associated with stalled or slow-moving replication forks, even under normal growth conditions. In addition, in both types of strains, almost all of the crossovers observed in sectored colonies had conversion events (3:1, rather than 4:0) indicating that the initiating DNA lesion occurred in S or G_2_. These results, taken together, suggest that the recombinogenic DNA lesions induced by replication stress likely occur at stalled or slow-moving replication forks.

On chromosome IV, both types of strains had a recombination hotspot located near three closely linked repeats, *HXT3*, *HXT6*, and *HXT7* (Figure 5). Although there are a number of interpretations of this observation, one possible explanation is that replication fork stalling at these repeats causes template switching, forming a recombinogenic secondary DNA structure. This same region is susceptible to deletion formation in the low Polδ strain, and is a source of extrachromosomal circles [55].

Despite the similarities described above, there are two substantial differences between the patterns of genomic instability induced by low Polα or Polδ. Low Polα had very small (about 2-fold) effects on the rate of small in/dels and base substitutions, whereas low Polδ elevates small in/dels about 500-fold, and base substitutions about 30-fold. The in/dels are flanked by short direct repeats, as has also been observed in strains with mutation in *POL3* [56], suggesting that these deletions are a consequence of DNA polymerase slippage. The low level of Polδ may lead to uncoupling of DNA polymerases at the replication fork, and increased slippage. Alternatively, a delay in recruitment to the Polα-synthesized primer may allow the formation of secondary structures in the single-stranded template, promoting slippage.

The elevated level of base substitutions in the low Polδ strain has been observed in two studies [13,31]. We suggest three possible explanations. First, a delayed recruitment of Polδ to the Okazaki fragment may result in long-lasting single-stranded regions that are prone to mutagenesis [57]. Second, low levels of Polδ may damage the replisome resulting in the recruitment of the error-prone Polζ polymerase [58]. Third, since Polδ is capable of removing errors introduced by Polα [59], a reduced level of Polδ may elevate the rate of Polα-induced mutations. We favor the second of these alternatives since Northam et al. [58] showed that the mutator phenotype associated with a point mutation in *POL3* was considerably reduced by loss of Polζ. Finally, we point out the amount of DNA synthesized by Polα is less than that synthesized by Polδ. Polδ is estimated to synthesize 5 to 10 times more DNA than Polα [2,60].

Another striking difference between the instabilities observed in the low Polα and Polδ strains is the ratio of LOH events to aneuploid events. This ratio is 3 (201/67) for the low Polα strain, and 0.86 (90/105) for the low Polδ strain [12,13]. This difference has a reasonably straightforward explanation. Whereas Polδ is required for efficient heteroduplex DNA extension during homologous recombination, Polα is not [61,62]. These results are consistent with the conclusion that both low Polα and Polδ have elevated levels of DSBs but these DSBs are more efficiently repaired by homologous recombination in the low Polα strain. By this model, lack of efficient repair in the low Polδ strain results in a high frequency of monosomes [13]. Since we find that low levels of Polα and Polδ result in trisomic strains, as well as monosomic strains, all aneuploidy is not the result of failure to repair a broken chromosome. Segregation of incompletely replicated chromosomes is another plausible mechanism.

In summary, although both low Polα and Polδ strains have very elevated genomic instability, a detailed analysis shows that the spectra of types of instability are not identical. This observation argues that assaying multiple types of instability is important in attempting to identify the causal genetic defect.

## 4. Relevance of Yeast Studies to Fragile Sites and Chromosome Alterations in Tumors

To what extent are our observations of genomic instability induced by low levels of DNA polymerase relevant to understanding genomic alterations that occur in tumors? An honest answer is that, at this moment, we do not know. We have shown, however, that the genomic alterations observed in our experiments share some properties with the events induced by replication stress in mammalian cells. First, the recombinogenic lesions in yeast preferentially occur at genomic regions that are associated with slow-moving or stalled replication forks. Fragile sites in mammalian cells tend to be late-replicating regions, and these regions are preferred sites for the copy-number variants induced in tumor cells [7]. Second, we show that a single genetic alteration produces multiple types of genomic alterations similar to those observed in tumors with the chromosome-instability (CIN) phenotype [63]. This property is consistent with the suggestion by Loeb [64] that an early step in carcinogenesis may be a mutation with a mutator phenotype. Third, we find that some of the mutations induced by low Polδ evolve the genome to allow faster growth of the strain containing the alterations (reduction in the level of rDNA and deletion of a repressor binding site in the *GAL* promoter). Thus, as in genetically unstable tumor cells, genetic changes that allow the cell to grow faster are generated.

Although tumor cells often exhibit LOH events, in general, the spectrum of the various classes of LOH events (gene conversions, mitotic crossovers, large deletions, ploidy changes) are not completely analyzed in tumors. It is clear, however, that most of the same types of LOH observed in yeast cells also occur in tumor cells, although the relative frequency of these classes of events is less certain [65,66].

The mechanisms by which LOH events are generated in mammalian cells have some similarities and some differences with those observed in yeast. In mammalian cells, for example, translocations are often the result of alternative NHEJ pathways rather than homologous recombination between non-allelic repeats (reviewed by Symington and Gautier [67]). In contrast, recurrent large deletions and duplications in human cells frequently reflect homologous recombination between non-allelic repeats [68] as observed in yeast.

The last issue that we will discuss is whether the genomic instability observed in tumor cells reflects replication stress imposed by low levels of the replicative DNA polymerases or associated co-factors. Cancer-related mutations have been described in the genes encoding DNA polymerases Polδ and Polε [69], although it is not clear that these mutations induce replication stress. It is likely that a long-lasting reduction in the level of replicative DNA polymerases in human cells would result in slow-growing cells that would be unlikely to give rise to a metastatic tumor. However, it is possible that a transient epigenetic inactivation of a gene encoding DNA polymerase or a co-factor would induce a transient high level of genomic instability. Reversal of this inactivation would restore a normal growth rate in a population of cells that would have many alterations, some of which may be cancer-promoting. Whether this mechanism operates for subset of cancers is, at the moment, unknown.

## 5. Summary

We showed that replication stress imposed by low levels of the replicative DNA polymerases Polα and Polδ result in very high levels of numerous types of genomic alterations. Our evidence suggests that these alterations are a consequence of a high level of recombinogenic DNA lesions likely induced at stalled or slow-moving replication forks. This mechanism has many features in common with that proposed for fragile sites in mammalian cells. Although most of the types of genomic alterations are in common in strains with low levels of either polymerase, there are also differences. One major difference is that high rate of base substitutions and small in/dels observed in strains with low Polδ but not low Polα. Lastly, we suggest the possibility that the genomic instability observed in some tumors could reflect the transient reduction in the amount of DNA polymerases or their co-factors.

## Figures and Tables

**Table 1:** Fold elevations of various types of genomic alterations (normalized to a wild-type level) in yeast strains with low levels of Polα and Polδ ^1^.

Relevant Phenotype	Rate of Mitotic Crossovers/BIR (Terminal LOH)	Rate of Gene Conversions (Interstitial LOH)	Large (>5 kb) Interstitial Duplications Plus Deletions	Large (>5 kb) Terminal Duplications Plus Deletions	Ploidy Changes and UPD (Monosomy, Trisomy, UPD)	Base Substitutions Plus Small (≤700 bp) in/dels
Low Polα	450	90	350 ^2^	250 ^2,3^	(13,000; 190; 1200) ^4^	2
Low Polδ	200	15	240 ^2^	90 ^2,3^	(16,000; 95; 800) ^4^	55 ^5^

^1^ In this table, we divide the rates of each type of genomic alteration observed in the low Polα and Polδ by the rates for the same type of alteration determined in the wild-type strain. ^2^ As discussed in the text, these numbers represent a minimum, since no large duplications/deletions were observed in the wild-type strain. ^3^ As discussed in the text, most of these terminal duplications and deletions were coupled, and reflect the formation of a translocation by the mechanism shown in Figure 6D. ^4^ The numbers in parentheses show the fold increases for monosomy, trisomy, and UPD (uniparental disomy) in the same order in which they are listed. The rate of UPD in the wild-type strain was calculated by multiplying the rate of UPD for chromosome V (determined in ref. [37]) by the number of chromosomes. ^5^ The low Polδ strain had a 30-fold elevation of base substitutions and a 500-fold elevation of small in/dels.
